# Translations from Foreign Exchanges

**Published:** 1883-04

**Authors:** 


### Translations from Foreign Exchanges.

BY O. STROINSKI, M.D., CHICAGO.

---

OSSIFICATION OF THE CHOROID.

The changes of tissue in the middle eye have been well studied but the changes in the choroid have not been fully described. Ossification of the choroid has been thought to be a result of changes in the vitreous lamella or in the capillaries of the former. Dr. Hoene of Warsaw, has examined two eyes enucleated by Dr. Galezowski, at Paris. One eye was affected with irido-choroiditis from traumatic influence. The middle of the eye-globe showed an osseous zone adherent to the sclerotic at the point of entrance of the optic nerve. In the anterior parts the sclera as well as the choroid could be easily detached from the osseous zone. The choroid which has a light appearance was more adherent to the sclera than to the osseous zone by the ciliary nerves which were well preserved. It was but at the divergence of the optic nerve that the two members were united so intimately to the osseous tissue that it was difficult to separate them. The ciliary body was absolutely free, and had no connection with the osseous tissue. The iris was free on its anterior aspect, but on its posterior surface was attached to the capsule of the lens. The lens was attached to the choroid by connective tissue which was easily detached with scissors. The same tissue was found in the middle of the choroid. The latter had a rough surface and showed on its aspect an osseous tissue. It could be divided into two layers, the posterior of which was hard and showed under the microscope the appearance of osseous tissue, the anterior part of the chorodea having a tissue of blackish color which could be easily removed with scissors. The different parts of the sclera and choroidea were examined under the microscope, the decalcification of the osseous tissue having been effected by a saturated solution of picric acid. Dissection of the sclera proved the fibrous tissue to be normal, but on the attachment to the optic nerve the fibrous tissue showed an intra-cellular infiltration and was evidently enlarged as it approached the choroid.

The adjacent choroid had not the elements constituting the normal tunica vascularis, but was entirely occupied by the new tissue. The large fibers composing the vitreous lamella were hypertrophied as also those of the amorphous tissue and of the small vessels. This tissue was colored by picrocarmine. The histological construction of the choroid differed essentially in its different parts. The ciliary body and the next attached parts of the choroid were not much altered here but the posterior parts showed great modifications. The alterations of the anterior parts were an hypertrophy of the walls of the blood-vessels with enlargement of the caliber. The cells contained less pigment but they were of normal size. The posterior parts of the choroid had no pigment and were changed to an amorphous mass with fine fibers containing small cells. Neither the chorio-capillaris nor the vitreous lamella could be distinguished, new tissue having taken its place. The vessels in this part were hypertrophied and showed cellular infiltration of the walls. This infiltration progressed forward, and the whole choroidea formed a fine layer intensely pigmented and surrounded by an embryonal layer which replaced the other layers of the choroid. The dissection in the osseous ring showed this to be a true osseous formation in form of osseous blades which united by prolongations and which had a meridional direction. Each blade was composed of osseous lamellæ which formed a circle around a central orifice, the canal of Harvey. The osseous corpuscles were disseminated between the singular lamellæ. Between the osseous blades was a connective tissue which formed by its fibers a small vessel. In the anterior parts the lamellous structure was less distinct. Between the small osseous blades there was a fibrous tissue with curved fibers which contained the same connective tissue interspersed with amorphous and fatty masses. The choroid was but slightly attached to the osseous tissue. In the posterior parts, the osseous blades were deposited in embryonal tissue and connnected with it by fibrillar prolongations. The nervous elements of the retina were lost and instead was found connective tissue with very fine fibers and globules. The corpus vitreum was changed to a layer of fine fibrillæ connected with the remnant of the retina. The osseous tissue was also dispersed to the inner side and the osseous blades transgressed freely the connective tissue.

The second eye-ball had been subjected to Critchett's operation, but, while the symptoms of sympathetic ophthalmia had attacked the other eye it had been enucleated. The ball had been preserved for ten months in alcohol and presented the form of a nut with flattening on the anterior surface traversed horizontally in the middle of the ball by a cicatrix which was the result of the operation. The margins of the cicatrix adhered to the contents of the globe. Beneath these adhesions, the sclera was easily detached on one side of the entrance of the optic nerve, on the other, on the macula. On the sclera was seen a very fine internal membrane containing pigment easily detached. This membrane was apparently the atrophied choroid and the ciliary body was hardly distinguishable. The iris was very transparent but intact. There were but traces of the lens as a natural cause of the operation. By slight traction the optic nerve was easily detached except on the macula lutea, which was whitish yellow in color and of osseous consistence. As to the formation of osseous tissue there are two different formations, one of connective changing directly into osseous tissue, the other by metamorphosis of cartilage by infiltration of cellular tissue with lime salts (metaplastic) which latter did not show the osseous blades of metaplastic formation. While the osseous tissue in these cases had a perfect lamellar structure it was the fibrillar tissue originating from embryonal tissue which formed the osseous tissue. It is therefore the opinion of the author, that the osseous tissue of the choroidea originates from embryonal tissue reproduced by excessive exudation from the choroid and which produces a new formation of the original tissue.—*Recueil d' Ophthalmologie.*

### On Strictures in the upper aerial duct caused by Scars.

In Volkmann's *Sammlung Klinischer Beiträge*, Dr. Jacobson publishes his experience with these strictures. They are caused mostly by traumatic influence. Syphilis is the source of a good many; and the author gives the autopsy of a woman, where the stricture was found in the fourth tracheal ring. Of infectious diseases causing strictures, typhoid fever, small-pox, diphtheria and glanders are named. There is stenosis of the upper part from its non-use, in persons who have undergone tracheotomy. Congenital stenosis is interesting from an anatomical standpoint. There are stenoses from granulations produced by ulcerative processes in the trachea, on the margin of ulcers. Stenoses may be provoked by defects and loss of substance, with consecutive reparation by scar tissue and shrinking, by thickening of the submucosa and of the perichondrium, caused by long-continued and deeply situated inflammation, in fractures and luxations by dislocations of the cartilages and their segments, by anchylosis of the joints of the ring and ary-cartilage, and by partial or total extirpation of the vocal cords. The cartilages being intact, there is but a slight narrowing, but otherwise these are the most indurated and narrowed strictures. Beneath the stricture the aërial duct is often dilated, with emphysema of the lungs. The strictures are single or multiple. They are membranous, ring-like, cylindrical or funnel-shaped. A slight stricture often gives no symptoms. Troubles in respiration are often more dependent upon such complications as swelling, accumulation of mucus, etc., than upon the degree of constriction. The first symptom of a stricture is very often a sudden attack of severe dyspnœa. In other cases, the trouble in respiration begins slowly, and that especially after traveling or long speeches. Respiration becomes gradually more difficult, and there are heard, even at a long distance, certain laryngo-tracheal noises. Here local pain is frequent, with croup and dysphagia, and the voice is more or less changed, according to the seat of the stricture. The diagnosis has to be made *by palpation of the larynx and trachea*, the laryngoscope being often of no use whatever. The seat of the stricture is of the greatest importance for the treatment of the case. After infectious diseases, the larynx is especially affected; in syphilis the stricture is mostly above the ring cartilage, and strictures of traumatic origin are found in the upper part of the throat. Strictures of the larynx change the voice considerably, and this is often noticed before the impeded respiration. Dysphagia, cough, and local pain are frequent. The trouble in respiration increases rapidly, and the larynx makes forced motions down to the jugulum and back. If the patient has undergone tracheotomy, the larynx has to be examined by a fenestrated canule, or by bougies or sounds. In strictures of the trachea or of the bronchi, the voice is clear, but weak; trouble in respiration increases gradually; stenosis of a main bronchus causes abolition of the respiratory murmur in the respiratory lung. The deeper the seat of the stricture, the more unfavorable the final result. The caliber and condition of the walls are of the greatest importance in the treatment.—*Correspond. Blatt f. Schweizer Aertze.*

### A Case of Large Abscess in the Brain.

The patient was a girl of seven years, and had suffered for two years from pains in the left temple, with caries of the squamous portion of the left temporal bone, and an aperture as large as a five-cent piece, but she had always been of an amiable character and fair intelligence. The opening was treated with iodoform. Afterward severe headache, with increased pulse, followed, and a high temperature, with vertigo, insomnia, and psychical excitability, with clonic contraction of the muscles. But she retained her consciousness, and even amidst the most severe pains in the head, she welcomed her relatives with joy. The diagnosis was acute meningitis, with diffusion of the inflammatory process. The treatment was without success. Epileptiform attacks, mydriasis, paralysis of the right extremities, and partial paralysis of the left side, with coma, followed. To relieve the pressure on the left cerebral hemisphere, a trepan was applied on the squamous portion of the left temporal bone, and 250 grms. of a dense pus withdrawn. A drainage tube was inserted, and a solution of phenic acid injected. In the afternoon, the girl was found sitting on her bed, without any symptoms of coma or paralysis. But the mydriasis coutinued, and the pulse became weak. On the next day an epileptiform attack set in, and vomiting followed. The flow of pus decreasing, a larger drainage tube was inserted, and the sac washed out. After two weeks aphasia followed, with slight paresis on the left side. After another week, hæmorrhage from the temporal aperture appeared, which was followed by general paralysis, coma and death. The autopsy showed, beside the large opening in the squamous portion of the temporal bone, adhesions of the meninges with the cranium, and a large aperture of the brain down to the inferior convolutions, and communicating with the left lateral ventricle. Complete softening of the cerebral cortex extended from the orbital portion to the frontal lobes, and from the superior parietal convolutions and the superior parietal sulcus to the left, and further from the margin of the ascendant parietal convolution to the right side. The brain itself formed a generally disorganized mass. The retained consciousness, and the continued use of language in a lesion of such extent, form a curious part of the case.—*Gazetta medica Italiana.*

THE SUBSEROUS FIBROID OF THE UTERUS.

In performing laparotomy for fibroma of the uterus, there has been made no differentiation as to whether the tumor was subserous, with a pedicle, or if it was intramural. This differentiation is at the present time of more importance than the question as to amputation in the body or cervix uteri. Theoretically, the removal of a subserous pedunculated tumor seems to be easier than the removal of an intramural tumor, but the mortality has been equal but for a short time. At present, the extirpation of pedunculated subserous tumors offers no difficulty, and only the pedicle remaining gives rise to complications. The symptoms of these tumors are very different; subserous tumors causing no hæmorrhages, while in submucous and intramural tumors the hæmorrhages are the main symptom. As mechanical results, there are flexions of the uterus, with pressure on the neighboring parts. They are especially serious in intramural tumors. The special kind of pain characterizes the region of development. In interstitial tumors, there is a pain of pulling down at the time of the menses; in submucous, there are labor-pains; in subserous, peritoneal pains. Ascites is frequent in submucous and interstitial fibroids as a cause of anæmia, produced by severe hæmorrhages; in subserous, by injury of the peritoneum. Peritonitis is frequent in the latter. A large-sized tumor provokes dyspnœa; of rarer occurrence are eclampsia, hæmato-hydro- and pyo-metra. Sterility is frequent in submucous and interstitial, rare in subserous tumors. The indications for laparotomy are, 1, in interstitial tumors, severe hæmorrhage; severe symptoms of pressure on adjacent parts, and dyspnœa of high degree. 2, in pedunculated subserous fibroids, severe pains; ascites; peritonitis; sterility; labor pains by pressure in dyspnœa. The prognosis in pedunculated subserous fibroids is favorable. The author, Dr. Boener, gives twelve cases, all of which recovered. Operations of a former date were unfavorable. The treatment of the pedicle is of great importance. Sometimes the adhesions connecting the uterus with the abdominal walls rupture suddenly, and these cases are favorable.—*Correspondenz Blatt f. Schweizer Aertze.*

SYPHILIS OF THE SPINAL CORD.

The anatomical changes of syphilis in the spinal cord are similar to those of the brain, but there has never been mentioned an anatomical change in the blood-vessels of the cord. The following case shows some interesting points in this respect. The patient was a woman forty-three years of age, who had always been healthy till her marriage. The husband had at the time of the marriage an ulcer on the genitals, and the woman had suffered from several abortions, with profuse hæmorrhage. During the last two years she became absent-minded, and her spirits were very low. This condition alternated with excessive excitement, which developed to mania errabunda. She died suddenly in collapse. The dura mater of the brain was thickened and turbid on the pons varolii, and this extended to the sulcus longitudinalis. There were adhesions between the dura and the brain substance. The arteries of the base of the brain, and especially the two arteries of the fossa Sylvii and the branches of the circulus arteriosus of Willis showed nodular concretions. The brain substance was generally anæmic, and on the corpus striatum there was a soft black spot. The cervical part of the spinal cord was hard. The inner aspect of the great arteries and veins showed microscopically a syphilitic degeneration. There were concretions in the walls of the vessels which diminished the caliber of the vessels in their diameter, and which occluded the lumen of the vessel to complete obstruction. The process of inflammation with exudation could be easily traced in every vessel. The obliterating process had been provoked by the pia mater, grossly infected with the syphilitic virus, and propagating itself in the veins and arteries in a special manner. There existed, besides the syphilitic lesion of the brain and the spinal cord, a chronic interstitial nephritis and osteosclerosis of the left caput femoris. —*Gazetta medica Italiana.*

### SURGERY À LA WAGNER.

Many musicians cannot comprehend the musical works written by Richard Wagner, and for many surgeons outside the German empire, the tasks undertaken by the surgeons of this enlightened country are difficult to understand. There was a virgin of twenty-seven summers, somewhat retarded in physical development from want of food and other necessities of life. This rather squat-figured lady came under the treatment of a quack, while suffering from irregularities in her monthly periods, who finished his abominable treatment by producing a prolapsus uteri et vaginæ. The lady went here and there to get relief from the swinging burden, but without avail. She then came into the hands of Dr. Kuhne, who found a common prolapsus, even without any dislocation on the part of the rectum or bladder. He tried some pessaries, but not being satisfied with their behavior, he performed episiorraphy. But even through the occluded vulva the vagina and uterus were seen dangling, much to the chagrin of the doctor. What now? he asked, and he extirpated both ovaries, the fallopian tubes, and a part of the fundus uteri, which latter he affixed to the abdominal walls with fine sutures, after having it covered well with the parietal peritoneum. This somewhat dangerous operation had the astonishing result that the prolapsus was seen dangling again in its old place, much to the chagrin of the doctor. He now became very angry, and he performed episiorraphy a second time, with apparently satisfactory results, and we have not heard of further operations on the prolapsus of the short-figured maiden.—*L' Union mêdicale.*

### SYPHILIS AND DEMENTIA PARALYTICA.

Tabes dorsalis and syphilis are in intimate connection; or rather, as recently proved, there are many cases of locomotor ataxy originating from syphilis. The relation between syphilis and dementia paralytica has not been sufficiently demonstrated. Dr. Oberstine has made examinations upon this point, and he found among 1,000 insane persons (660 males and 340 females), 175 affected with dementia paralytica. Syphilis was found among the whole number 73 times. Paralysis and syphilis were observed in the same person 37 times, *i. e.*, 21.6 per cent. of male persons affected with paralysis had been syphilitic, and in 51.4 per cent. of syphilitic persons progressive paralysis ensued. Of all the other 825 patients, but 35 (4.1 per cent.) were affected with syphilis. The number of patients suffering from syphilis and dementia paralytica was, therefore, five times greater than that of other insane persons suffering from syphilis. The time of syphilitic infection and of the developing dementia varied from six to seven years. Dementia paralytica is often regarded as a chronic periencephalitis, followed by sclerosis and atrophy of the dura mater, without its further consequences. The syphilitic appearances of the brain are, 1, a localized; 2, a more diffuse form. The former is characterized by the apperance of tumors; the latter affects those parts supplied abundantly with blood-vessels, as the dura mater and pia mater. The antisyphilitic treatment of these insane patients has always been followed by rapid improvement.—*Monatschrift f. pract. Dermatol.*

### TETANUS IN LYING-IN WOMEN.

K. H., twenty-three years of age, entered the lying-in hospital at Vienna, and was there delivered by craniotomy. On the third day the pulse was 86; temp. 88; tongue dry and red, and cheeks feverish. Patient complained of weakness. A short time after, the first attack set in. On the upper extremities the thumbs were in adduction, and grasped in the palm of the hand; the other fingers only flexed in the metacarpo-phalangeal joints. The forearms were semi-flexed, the hands slightly curved at the wrist, the upper arms adducted. Active and passive motion were impeded by contraction of the muscles. The lower extremities were flexed at the hips and knee-joints, the upper limbs adducted, the muscles of the calf contracted. Any attempt to extend the lower extremities was accompanied by severe pains. By compression of the large blood-vessels of the extremities, the phenomena above described could be easily produced, and on irritation of the nervus facialis by palpating the same, the whole group of the facial muscles was contracted. Bromide of potassium and hypodermic injections of morphine easily relieved the patient. This disease, which is called tetania by Trousseau, has been lately observed quite often. Contractions of the muscles of the trunk have also been seen in these cases. The attacks are repeated in different intervals, and it seems that after a long protracted confinement, the attacks are more severe, and they last longer. The disease is not followed by death or severe consequences.—*Wiener Med. Wochenschrift.*

CANCER OF THE UMBILICUS.

N. W., fifty years of age, entered the hospital October 15, 1880. He complained of excruciating pains in the region of the umbilicus. In the month of November, of the same year, he had zoster in the course of the nervus obturatorius. The patient became emaciated. In the region of the umbilicus there was a tumor of the diameter of two cm. It was of a bluish color, and a little ulcer there separated a serous fluid. The tumor was hard, and the induration around the tumor extended to two cm. in the circumference of the umbilical cicatrix. The diagnosis was cancer of the umbilicus, and the tumor was extirpated antiseptically, the abdominal wall healing per primam. The patient died from cancer of the rectum one year afterward. Under the microscope, the tumor had all the characteristic forms of a cancer. It was covered by a cutis which showed certain tuberous elevations, similar to those of papilloma. The superficial layer was formed by a stroma of connective tissue, with numerous spaces of different form. The connective tissue was of the alveolar structure, and among the alveoli were interspersed epithelial cells. In the middle part of the tumor, the epithelial cells were more frequent and larger (giant-cells). In the inferior part there were numerous vessels with transverse nuclei. There were also numerous cylindrical epithelial cells.—*Giornale internazionale delle scienze mediche.*

### A CASE OF POISONING BY STRYCHNINE TREATED WITH CHLORAL HYDRATE. RECOVERY.

The patient, a girl twenty-three years of age, had taken forty centigrm. of strychnine with suicidal intent. An emetic had been given an hour and a half after taking the strychnine, and tannic acid in large doses had also been employed, without relieving the convulsions. The girl had first clonic convulsions, which were followed by tetanus. Forty convulsions had been counted in three hours. Five hours afterward there was complete opisthotonos, with pleurosthotonos, followed by tetanic spasm. An hypodermic injection of .001 pilocarpine was made, and was followed by profuse salivation and perspiration. Chloral hydrate, 12 grms., was administered by the mouth, and as the convulsions did not cease, .030 chloral hydrate was injected hypodermically every half hour. Three hours afterward, another injection of .001 pilocarpine was applied. The body was sometimes in a state of complete rigidity, and the pulse changed from 90 to 120. The lower limbs were in perfect extension, and flexion was impossible. The convulsions stopped twenty-four hours after taking the strychnine, but they reappeared at intervals. Chloral hydrate was applied for the next twenty-four hours, and the doses given amounted to over 100 grm., mostly injected subcutaneously.—*Archives générales de médecine.*

### SYPHILIS OF THE PHARYNX.

The patient, a woman thirty-two years of age, lost her appetite and became emaciated after the birth of her first child. She went to Esplugo de Francoli to use the iron baths, and afterward was treated by hydrotherapeutics, but without result. The mucous membrane of the gums and of the conjunctiva became pale, and there was general atrophy of the muscular system. A slight fever, with night sweats and a high pulse, followed, and difficulty in swallowing was noticed. The symptoms indicated general progressive anæmia. The gums and the tonsils were free from any affection, and only on the posterior part of the pharynx a circular spot was visible, of the size of a dime. It looked like the product of a chronic catarrh. The superficial layer of this spot having been removed by a brush, its true character was easily detected. There was hyperæmia of the mucosa of the larynx, which extended into the Eustachian tube, with diminished hearing and pains. There was profound perturbation of nutrition, but no visible symptoms of general syphilis. Mercury and iodide of potassium effected a speedy cure. The case is remarkable, in that a single spot on the pharynx, without other symptoms of syphilis, reduced the patient to a mere skeleton.—*La Independencia medica Barcelona.*

ANATOMICAL CHANGES IN THE BRAIN IN INFECTIOUS DISEASES.

The tunica interna of the cortical layer shows here a granular fatty deposit, and tumefaction of the endothelial cells. In some cases of typhoid fever and septicæmia, there are colloid spots in the adjacent parts of the vessels. The gangliar cells are entirely changed in the layer of the large cells. In examining the dissections from the periphery to the inner parts, there is found tumefaction of the protoplasm. The pyramidal cells become round or irregular. The fatty granular degeneration is differently developed. In the most advanced stage of degeneration, there are found large nuclei in the intercellular spaces, surrounded by granular protoplasm. In minute examination, some nervous elements are to be found degenerated, and it is probable that gangliar elements have been multiplied by direct division of the nuclei. The anatomical changes in the medulla oblongata are constant. From all the nuclei of nervous origin, and especially those of the pneumogastric nerve, originate normal multipolar cells, round, oval and irregular, also with turbid protoplasm, and there is sometimes a distinct nucleus with a short prolongation. Most cells are smaller than usual. Parenchymatous alterations are also found in the brain.—*Gazetta medica Italiana.*

### A Case of non-Tubercular Basilar Meningitis.

A girl, seven years of age, complained of headache and general tremor of the body. Temp. 39.8 R.; pulse 100. Sensorium free; headache most intense on motion of the body. Pupils dilated, reacting well; spleen enlarged; constipation; respiratory organs normal. On the 11th of April, strabismus convergens, with small pupils; facial paresis of the left side; pulse regular; sensorium free. Four days afterward, pupils dilated; abdomen retracted; pulse irregular, with repeated vomiting, and strabismus. At four o'clock in the afternoon, very restless; unconscious; frequent vomiting. On the 26th, complete abolition of consciousness; very much emaciated; splenic tremor persistent. On the 30th, difficulty in swallowing, and death. The course of the disease was fifty-four days, with irregular fever. The autopsy showed basilar meningitis, with internal hydrocephalus and softening of the ventricle. The pia mater was intact; no tubercles found. The diagnosis first indicated tubercular meningitis, but soon the course of the disease proved the case to be of non-tuberculous character.—*Gazetta medica Italiana.*

### Paralysis from Hypodermic Injections of Ether.

A. B., twenty-one years of age, had varioloid, and two injections of ether were made on the posterior region of the left forearm (what for?) The next day she complained of severe pains at the point of injection, and three fingers of the left hand lost the power of motion. The pain continued for a week, and also the paralysis of the fingers. The left hand lay in semipronation, the index finger but slightly flexed, while the thumb and middle finger were thrown entirely into the palm of the hand, the other fingers being normal. The flexion of the forearm was perfect, and in this movement there could be felt a contraction of the supinator longus muscle. The electrical exploration showed not the slightest contraction of the extensor communis and abductor of the thumb. Sensibility was intact. The left forearm measured one cm. less than the right arm. Continued applications of the galvanic current relieved the suffering girl.—*Archives générales de médecine.*

### AN ADENOID TUMOR OF THE HARD PALATE EXTIRPATED WITH LOWERING OF THE HEAD.

The patient, twenty-four years of age, was the wife of a teacher, who noticed four years ago a small growth on the right side of the palate. At present there was seen a round tumor, of the size of a walnut, reaching forward to the first grinder. There was a transverse scar, resulting from a former incision. At this part the tumor was soft, but at the base of the tumor there was a hard shell, which terminated on both sides with a sharp point. The patient was operated upon with downward hanging head, and the bleeding was so profuse as to protract the otherwise easily performed operation. The hard palate was scraped out with a sharp spoon. Dr. Weinlechner, who performed the operation, has seen *profuse hæmorrhage in all operations with lowered head.* The tumor was an adenoma, with elements of carcinoma. —*Med. Chirurg. Centralblatt.*

### RETENTION OF THE PLACENTA FOR TWO YEARS.

Dr. Raugé reported this case in the Société Médicale de l'Aix. The woman was thirty years of age; primipara, and had an abortion in the second or third month of pregnancy, but no physician attended. Severe hæmorrhage persisted for months. For the period of one year afterward the woman suffered from intense pains in the abdomen, and this was accompanied by profuse leucorrhœa. She then suffered from diarrhœa and dysentery. Nineteen months after the first symptoms, the doctor was called, and found the woman in labor-pains. Two hours later, an entirely putrefied placenta was expelled, and a part of the umbilical cord was yet attached to it. Metritis followed, with hæmorrhages which continued for a year. The woman asserts that she never had any connection with her husband from the first attack, and the doctor believes the placenta to be nearly two years old. (What a placenta and what a cord !—TR.)—*Lyon Médicale.*

OBITUARY.—Dr. D. O. Farrand, an eminent surgeon of Detroit, Michigan, died of congestive apoplexy March 18, 1883, aged forty-five.

